# Malaria among schoolchildren in urban, peri-urban and rural settings in Eastern Ethiopia: repeated cross section study

**DOI:** 10.1186/s12936-025-05591-4

**Published:** 2025-10-09

**Authors:** Hailu Merga, Teshome Degefa, Zewdie Birhanu, Ephrem Abiy, Ming-Chieh Lee, Guiyun Yan, Delenasaw Yewhalaw

**Affiliations:** 1 Department of Epidemiology, Institute of Health, Jimma University, Jimma, Ethiopia; 2 School of Medical Laboratory Sciences, Institute of Health, Jimma University, Jimma, Ethiopia; 3 Departement of Health, Behavior, and Society, Faculty of Public Health, Jimma University, Jimma, Ethiopia; 4 Abt Associates, PMI VectorLink Ethiopia Project, Addis Ababa, Ethiopia; 5 Program in Public Health, University of California at Irvine, Irvine, USA; 6 Tropical and Infectious Diseases Research Center, Jimma University, Jimma, Ethiopia

**Keywords:** Epidemiology, Malaria, Urban, Peri-urban, Rural, Schoolchildren, Ethiopia

## Abstract

**Background:**

Despite malaria control efforts, schoolchildren continue to exhibit high malaria prevalence. While multiple studies report varying malaria prevalence among schoolchildren across Africa, there is an evidence gap in comparing malaria prevalence across urban, peri-urban and rural settings, particularly in the context of changing transmission dynamics due to *Anopheles stephensi* spread in Africa. Hence, this study aimed to assess epidemiology of malaria among schoolchildren in different eco-epidemiological settings during high and low transmission seasons in Eastern Ethiopia.

**Methods:**

A repeated cross-sectional study was conducted in November 2023 and May 2024 among selected primary schoolchildren in Eastern Ethiopia. Sample size was calculated using a single population proportion formula and design effect was considered. Simple random sampling technique was employed to select schools and students. Finger prick blood was collected, thin and thick blood smears were prepared, stained with 10% Giemsa and examined microscopically. Data on sociodemographic characteristics, and other determinant factors were collected using a pretested structured questionnaire. Data were collected using Open Data Kit (ODK) software and analyzed using Stata software. Binary logistic regression analysis was conducted to assess associations between independent variables and malaria infection.

**Results:**

The prevalence of malaria among schoolchildren during high and low transmission season was 5.1% (95% CI 3.84, 6.7) and 2.24% (95% CI 1.48, 3.37) respectively. With regard to the ecological settings, the prevalence of asymptomatic malaria among schoolchildren during high transmission in urban, peri-urban and rural were 7.3%, 4.97%, 2.9% (P = 0.007), respectively. Similarly, the prevalence during low transmission season in urban, peri-urban and rural were 3.5%, 1.2%, and 2.04% (p = 0.3) respectively. *Plasmodium falciparum* was the dominant species in all school settings, both during high (83%) and low transmission (59%) seasons. Non-use of ITN (AOR 2.2; 95% CI 1.2, 3.99) and presence of under-five children (AOR 1.9; 95% CI 1.1, 3.38) in the household were identified as predictors of malaria infection during the high transmission season. Similarly, the marital status of the child’s household head (AOR 4.4; 95% CI 1.4–13.8) was associated with malaria infection during the low malaria transmission season.

**Conclusion:**

Malaria intervention efforts should target all school settings, with particular emphasis on urban and peri-urban areas. There is a need to strength the use of insecticide-treated nets, focusing on households with under-five children, and malaria interventions should consider household socio-demographic contexts to reduce the malaria burden effectively.

## Background

A world free of malaria is a major goal of global health [1]. Malaria remains a major public health problem in the tropical countries of the world, especially in sub-Saharan Africa. It continues to have an impact on people's health and livelihoods globally, despite the fact that it is preventable and treatable. *Plasmodium falciparum* and *Plasmodium vivax* are the two most prevalent malaria parasites in Africa, and *P. falciparum* the most dangerous [2].

The World Health Organization (WHO) world malaria report 2024 indicated that an estimated 263 million malaria cases were reported globally—an increase of 11 million cases from 2023 report. The WHO African Region remained the most affected, accounting for 94% of all cases [2, 3]. Ethiopia is one of the sub-Saharan African countries where two malaria species, *P. falciparum* and *P. vivax*, co-exist which makes malaria control more complicated [4, 5].

The invasion and spread of *Anopheles stephensi,* a vector that thrives in artificial water containers and can withstand high temperatures during dry seasons, presents additional difficulties for the control of urban malaria in Africa. Malaria outbreaks are more likely to occur in African cities, especially those in the Horn of Africa, due to its adaptability, rising insecticide resistance, and unplanned urbanization [6–8].

Malaria remains a major public health concern among urban school-aged children, with asymptomatic cases serving as hidden reservoirs that undermine control and elimination efforts [9–14].

Malaria prevalence among schoolchildren varies widely, with studies reporting rates of 5.1% from southwestern Ethiopia [9], 6.8% from Northwest Ethiopia [15], 12.8% from Yemen [13], 21.6% and 38.1% from Tanzania [12, 16], 33% from DRC [17], and 30% from Rwanda [18]. Peak malaria transmission in Ethiopia takes place from September to December, following the main rainy season (June to August), and the second minor transmission takes place from April to June, following a short rainy season (February to March) [19, 20]. Although malaria is more common in rural regions, urban schoolchildren are nonetheless at significant risk, especially in peri-urban areas and those with inadequate control measures [21]. Evidence indicates that environmental factors, travel history, parental education, and ITN use influence urban malaria risk. Although bed net utilization is generally higher in urban areas, gaps in coverage and consistent use still persist [12, 15–18, 22–24].

*Plasmodium* infections among schoolchildren not only hinder elimination efforts but are also linked to long-term health impacts, including increased school absenteeism and impaired academic performance [9, 25]. Several studies have assessed malaria among schoolchildren in Africa, but since spread of *An. stephensi* in the Horn of Africa, no study has assessed and compared malaria prevalence among urban, peri-urban and rural schoolchildren during both high and low transmission seasons. Hence, this study aimed to assess and compare the prevalence of malaria among schoolchildren in urban, peri-urban, and rural settings during both high and low transmission seasons in *An. stephensi* hotspot area in Eastern Ethiopia. The findings will help policymakers better understand the burden of urban malaria, particularly in the context of the spread of *An. stephensi*.

## Methods

### Study design and setting

**Fig. 1:**
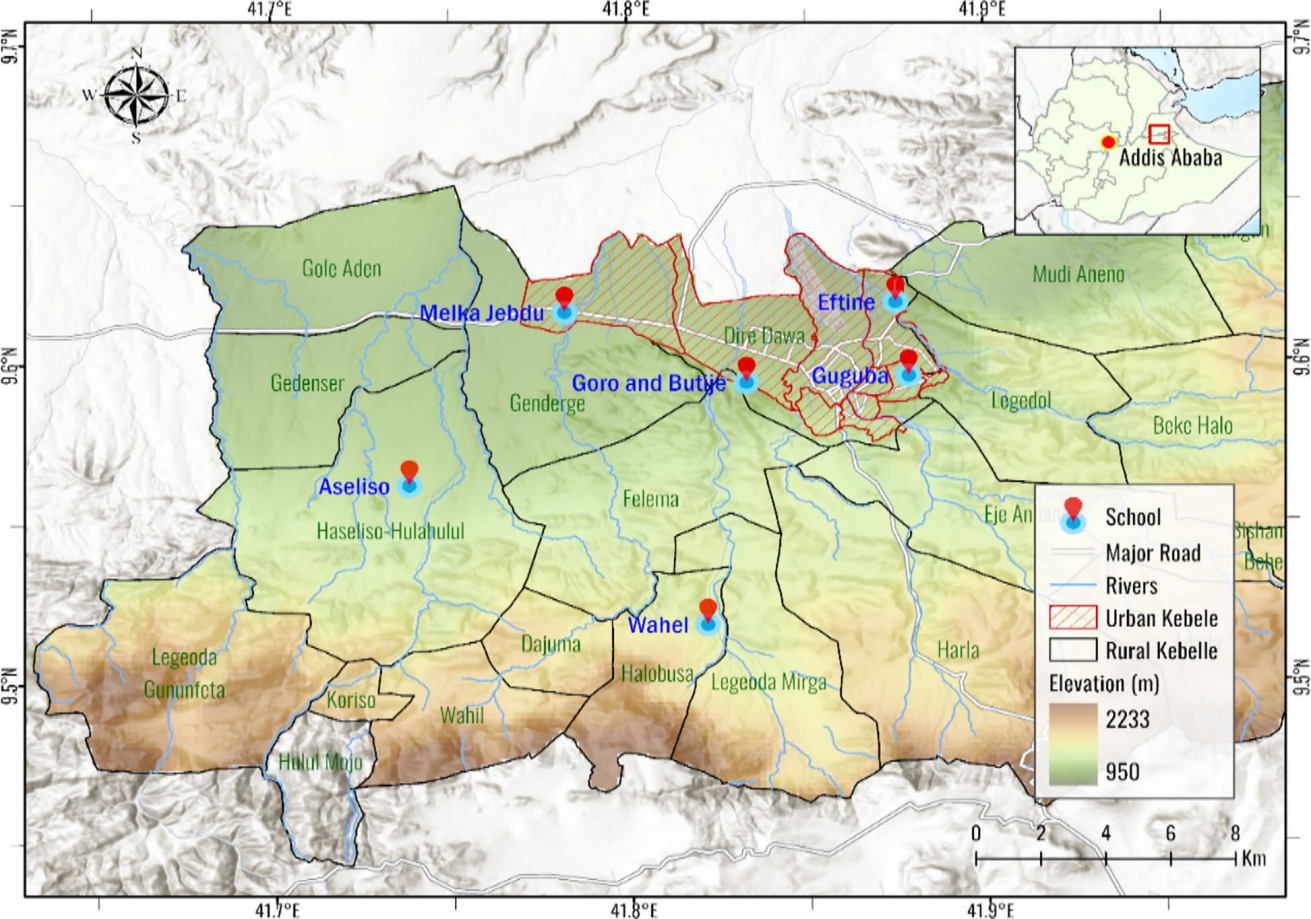
Map of the study area (Dire Dawa, 2024)

A repeated cross-sectional study was conducted in November 2023 and May 2024 in Dire Dawa administration, located in the eastern part of Ethiopia, at 515 km away from Addis Ababa, the capital of Ethiopia. The study were conducted during two different malaria transmission seasons: the first study took place in the high transmission season in November, and the second study was conducted in the low transmission season in May. Dire Dawa is one of the two city administrations in the Federal Democratic Republic of Ethiopia with about 550,642 residents. It is located at 9° 36′ N 41° 52′ E along the Ethio-Djibouti transportation corridor. It comprises urban centers as well as surrounding peri-urban and rural kebeles. Dire Dawa is an eastern free trade zone and was the epicenter for the recent outbreak of malaria associated with *An. stephensi* [26, 27]. The area experiences a warm, dry climate with low precipitation, averaging 624 mm annually, and temperatures ranging from 19 °C to 32 °C. Malaria incidence has historically been low, with an annual parasite clinical incidence of fewer than 5 cases per 1000 people between 2014 and 2019. The disease shows strong seasonality, peaking between August and November, with coexisting infections of *P. falciparum* and *P. vivax* [27]. There are two public hospitals, six private hospitals, 16 health centres and 59 private clinics in the city. There are 84 public and private schools in Dire Dawa administration (Fig. 1).

### Population

All public primary schoolchildren at the age group between 6 and 15 years from urban, peri-urban and rural were the source population while all selected public primary schoolchildren available during data collection were the study population. Children and/or their parents, guardians, or caregivers who were not willing to be included in the study and absent during study period were excluded. Students treated with anti-malaria drugs within one month prior to the survey were also excluded from the study.

### Sample size and sampling technique

The sample size was calculated using single population proportion formula by using the following assumptions: 95% CI 2% margin of error, 6.8% prevalence of malaria from previous study from North west Ethiopia [15] and 1.5 design effect. Finally, with the addition of 10% non-response rate, 1004 sample size was used in this study. The lists of all primary schools in the catchment areas of this study were obtained from the Dire Dawa education bureau. The schools with equivalent number of students were categorized into urban, peri-urban and rural schools. Then, two schools from each cluster were selected using simple random sampling. This is based on this research hypothesis to compare the prevalence of malaria in ecological zones (urban, rural and peri-urban). The calculated sample size was then proportionally allocated to each school and then to each grade and section. Finally, simple random sampling method was employed to select individual students from each section.

### Data collection tool and procedure

Data were collected with Open Data kit (ODK) software through face-to-face interviews. The interviews were conducted at the school compound. Before data collection, written informed consent was obtained from parents/guardians during school meetings that were specifically organized to explain the objectives and procedures of the study. In addition, verbal assent was obtained from all participating children aged 6–15 years prior to the interviews. Data collection tool was developed after reviewing relevant and related literature [11, 15, 24, 28]. The questionnaire was translated to both Afan Oromo and Amharic languages and back translated to English to check for consistency. Data was collected by trained and experienced data collectors/interviewers and laboratory technicians. To identify ownership and use of insecticide-treated nets (ITNs), the nets were brought to the schools during data collection. A sample insecticide-treated net (ITN) was taken to the school as a reference model and asked students to confirm whether they had a similar net at home and whether they had used it the night before the survey.

### Blood sample collection and laboratory analysis

Finger-pricked blood sample was collected from each student, and both thick and thin blood smears were prepared on a frosted-ended microscopic slide. Then, the thin film was fixed with absolute methanol. Finally, the slides were stained with 10% Giemsa for 10 min, air dried, and examined microscopically by an experienced microscopist. The slides were considered negative if no parasite was seen after examining 100 high power fields. To ensure the quality of data, two days training was given for data collectors team (interviewers, laboratory technologists and supervisors). The training covered how to collect data with ODK software, data collection approach, how to handle the sample and how to conduct laboratory investigations following standard operation procedures (SOP). As part of quality control, all positive blood smear slides and 10% of the negative slides were re-examined by another blinded senior laboratory technologist.

### Data processing and analysis

STROBE checklist [29] was used to analyse and report the data. Data was downloaded from the server and appropriate cleaning was done before both descriptive and inferential analysis using Stata version 16 software (Stata Corp. College Station, TX., USA). Binary logistic regression analysis was performed to determine association between the independent variables and malaria infection. All variables associated with malaria infection at a P-value ≤ 0.25 on the bivariable analysis were entered into the final model, multivariable logistic regression analysis. Finally, the predictors of malaria infection among schoolchildren were declared with Adjusted Odds Ratio (AOR), 95% Confidence interval and p-value < 0.05. A multicollinearity test and goodness of fit of the model were done using variance inflation factor (VIF) and Hosmer–Lemeshow test, respectively.

## Results

### Sociodemographic characteristics of respondents

Of the 1004 sample size calculated, 948 (94.4%) and 983 (97.9%) children participated in the study during the high and low transmission seasons, respectively. More than half of the schoolchildren, 595 (60.5%) during the high transmission season and 412 (43.5%) during the low transmission season, were in the 9 to 11 year age group. Similarly, the majority were males (56.8%), and 608 (61.9%) were in grade three or below. A large majority of the respondents indicated that the household head was married, with 886 (93.5%) during the high transmission season and 941 (95.7%) during the low transmission season. The majority of those children's mothers didn't attend formal school both during high (52.85%) and low transmission seasons (36.6%). Similarly, about one-third (33.3%) and more than one-fourth (27.9%) of children's fathers didn’t attend formal school during high and low transmission seasons, respectively. With regard to living arrangements, in both high (91.46%) and low transmission seasons (85.96%), the majority of schoolchildren were living with both their mothers and fathers (Table 1).

**Table 1:** socio-demographic characteristics of schoolchildren in Eastern Ethiopia, 2025

Variable		Variable category	High transmission season (%)	Low transmission season (%)
Age		< = 8 years	229 (24.2)	280 (28.5)
		9–11 years	412 (43.5)	595 (60.5)
		12–15 years	307 (32.4)	108 (10.9)
Sex		Male	533 (56.2)	558 (56.8)
		Female	415 (43.8)	425 (43.2)
School	Rural	Aseliso	152 (16.1)	160 (16.3)
		Wahel	160 (16.9)	183 (18.6)
	Urban	Guguba	164 (17.3)	156 (15.9)
		Goro and Butiji	151 (15.9)	162 (16.5)
	Peri-urban	Eftine	163 (17.2)	151 (15.4)
		Melka Jabdu	158 (16.7)	171 (17.4)
Grade		< = 3 grade	528 (55.7)	608 (61.9)
		4–5	280 (29.5)	357 (36.3)
		> = 6	140 (14.8)	18 (1.8)
Family size		< = 6	491 (51.8)	541 (55.0)
		> 6	457 (48.2)	442 (45.0)
Marital status of the Household head		Single	8 (0.8)	2 (0.2)
		Married	886 (93.5)	941 (95.7)
		Widowed	14 (1.5)	6 (0.6)
		Divorced	40 (4.2)	34 (3.5)
Mother’s educational status		Didn`t attend formal school	501 (52.9)	360 (36.6)
		Only read and write	13 (1.37)	16 (1.6)
		Primary education	258 (27.2)	281 (28.6)
		Secondary education	100 (10.6)	165 (16.8)
		Higher education	31 (3.3)	36 (3.7)
		Mother died/don`t know mother	10 (1.1)	5 (0.5)
		Don`t know	35 (3.7)	120 (12.2)
Father`s educational status		Didn`t attend formal school	316 (33.3)	274 (27.9)
		Only read and write	12 (1.3)	13 (1.3)
		Primary education	300 (31.7)	216 (21.9)
		Secondary education	151 (15.9)	223 (22.7)
		Higher education	80 (8.4)	85 (8.7)
		Mother died/didn`t know mother	35 (3.7)	26 (2.6)
		Don`t know	54 (5.7)	146 (14.9)
Mother's occupation		Farmer	44 (4.6)	55
		Merchant	299 (31.5)	378
		Governmental/NGO employee	48 (5.1)	36
		House wife	535 (56.4)	488
		Didn`t know mother/mother died	10 (1.1)	8
		Don`t know	12(1.2)	16
Father's occupation		Farmer	352 (37.1)	369 (37.5)
		Merchant	396 (41.8)	361 (36.7)
		Government/NGO employee	120 (12.7)	127 (12.9)
		Driver	22 (2.3)	7 (0.7)
		Didn`t know father/father died	35 (3.7)	47 (4.8)
		Don`t know	23 (2.4)	72 (7.3)
Place of residence		Rural	318 (33.5)	338 (34.4)
		Peri-urban	324 (34.2)	331 (33.7)
		urban	306 (32.3)	314 (31.9)
Living with		Either father or Mother	53 (5.6)	97 (9.9)
		Both mother and father	867(91.5)	845 (85.9)
		Sister/brother	17 (1.8)	40 (4.1)
		Others**	11 (1.2)	1 (1.0)
Number of under 5 children in the HH		No child	405 (42.7)	455 (45.9)
		1 child	323 (34.1)	397 (40.4)
		2 children	188 (19.8)	112 (11.4)
		3 and above children	32 (3.4)	23 (2.34)

Others** alone/another relatives/caregiver

### Prevalence of malaria among schoolchildren in urban, peri-urban and rural

**Fig. 2:**
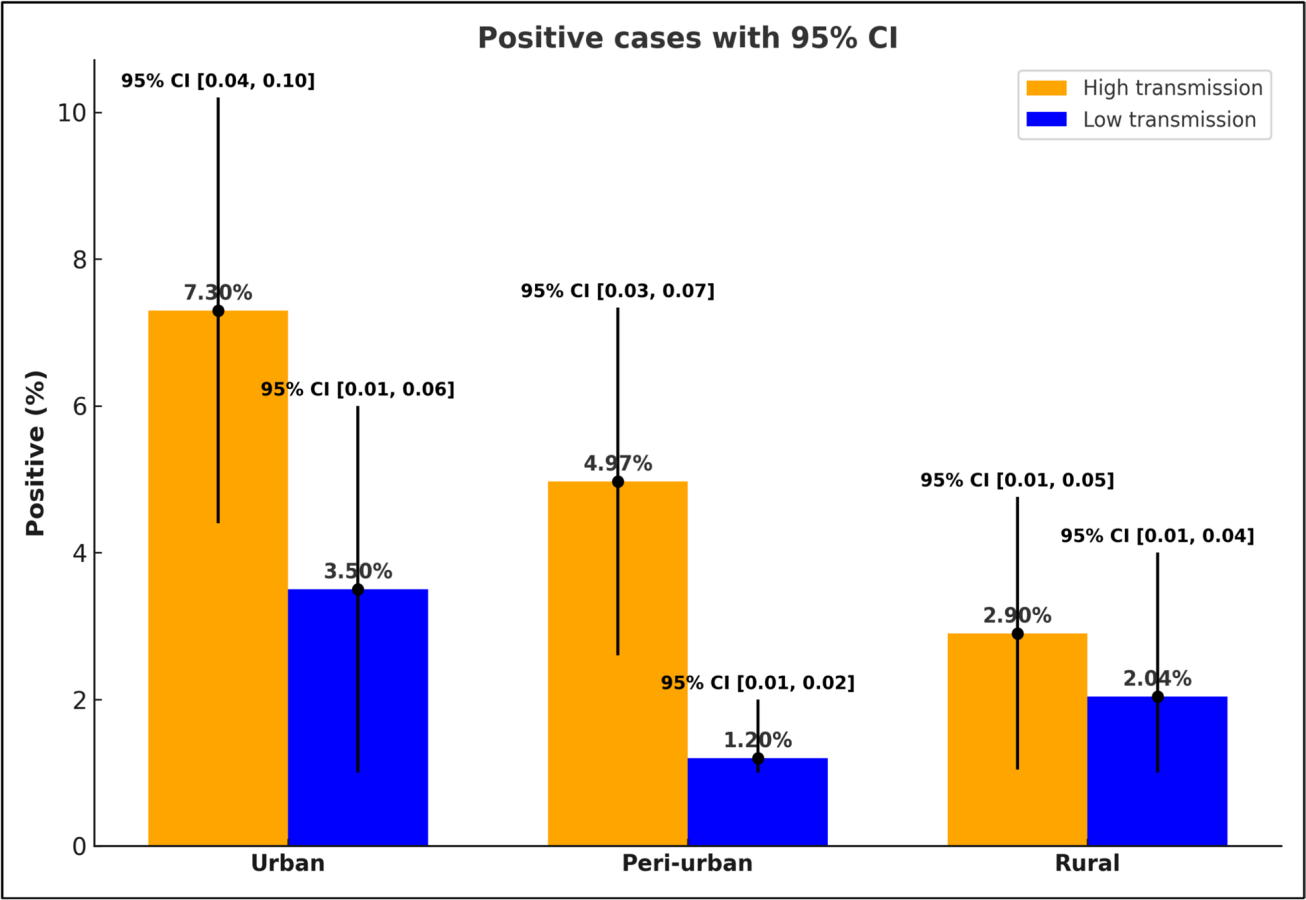
prevalence of malaria during high and low transmission seasons

The prevalence of asymptomatic malaria among schoolchildren during high transmission in urban, peri-urban and rural were 7.3%, 4.97%, 2.9% (P = 0.007), respectively. Similarly, the prevalence during low transmission season in urban, peri-urban and rural were 3.5%, 1.2%, and 2.04% (p = 0.3), respectively. The overall prevalence during high transmission and low transmission seasons were 5.1% (95% CI 3.84, 6.7) and 2.24% (95% CI 1.48, 3.37), respectively (Fig. 2 and Table 2).

**Table 2:** Prevalence of malaria by residence during high and low transmission seasons

Residence	Microscopic results					
	During high transmission			During low transmission		
	Positive (%)	Negative (%)	Total (%)	Positive (%)	Negative (%)	Total (%)
Urban	23 (7.3%)	291 (92.7%)	314 (100%)	11 (3.5%)	307 (96.5%)	318 (100%)
Peri-urban	16 (4.97%)	306 (95.03%)	322 (100%)	4 (1.2%)	318 (98.8%)	322 (100%)
Rural	9 (2.9%)	303 (97.11%)	312 (100%)	7 (2.04%)	336 (97.96%)	343 (100%)

**Fig. 3:**
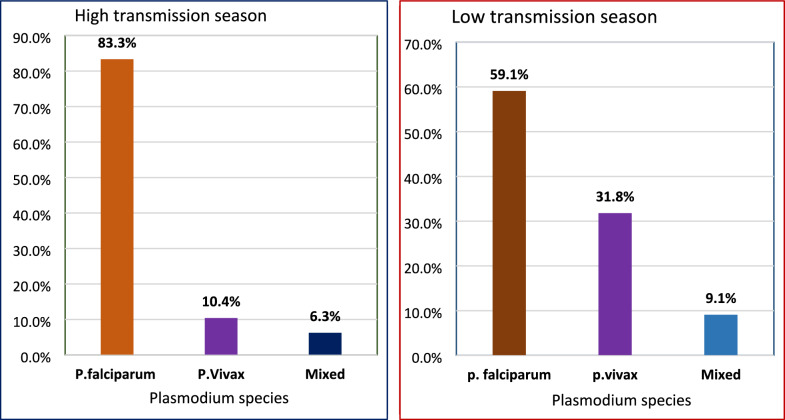
Proportion of Plasmodium species detected by microscopy during high and low transmission season

Regarding malaria species, *Plasmodium falciparum* was the dominant species during both seasons. During the high transmission season, of the 48 malaria cases, 40 (83.3%) were *P. falciparum*, 5 (10.4%) were *P. vivax*, and the remaining cases were mixed infections. Similarly, during the low transmission season, of the 22 cases identified, 13 (59.1%) were *P. falciparum*, 7 (31.8%) were *P. vivax*, and the remaining cases were mixed infections (Fig. 3).

### Housing condition and ITN utilization

During high transmission season, less than one third (32.91%) of the schoolchildren reported that they had ITN, while over one third (40.7%) reported during low transmission season that they had ITN. Similarly, among those who had an ITN at home, nearly two-thirds (63.5%) reported sleeping under it the night before the survey during the high transmission season, compared to about one-third (38.7%) during the low transmission season. More than one third of schoolchildren (42.2%) reported having two bedrooms during the high transmission season, while nearly half (48.4%) reported having a single bedroom during the low transmission season. Similarly, nearly two-thirds (64.9%) and more than three-fourths (77.4%) of respondents' homes had cement or ceramic floors during the high and low transmission seasons, respectively. Almost all respondents (99.2%) in high transmission, and about two thirds (66.4%) during low transmission season, reported that their house roofs were made of steel (Table 3).

**Table 3:** Housing condition and ITN utilization among schoolchildren in Eastern Ethiopia

Variable and category	High transmission season		Low transmission season	
	Frequency	Percentage	Frequency	Percentage
Had ITN/LLINs in the house				
Yes	312	32.9	583	40.7
No	636	67.1	400	59.3
Slept under the bed net last night				
Yes	198	63.5	226	38.7
No	114	36.5	357	61.2
Number of bed rooms				
One	293	30.9	476	48.4
Two	400	42.2	345	35.1
Three	185	19.5	93	9.5
Four and above	70	7.4	69	7.01
Floor material of their house				
Soil	333	34.1	177	18.0
Cement/ceramic	615	64.9	761	77.4
Wood and others	–	–	45	4.6
Roof material of their house				
Steel	940	99.2	653	66.4
Mud	2	0.2	272	27.7
Cement and stone	3	0.3	58	5.9
Others**	3	0.3	–	–

^**^Wood, Grass

### Determinants of malaria infection among schoolchildren

To identify the predictors of malaria infection among schoolchildren, bivariable logistic regression analysis was performed to select the candidate variables for the final model. Accordingly, during the high malaria transmission season, the following were identified as candidate variables: school location, child’s place of residence, ITN ownership, sleeping under an ITN the night before the study, number of under-five children in the household, and number of bedrooms. However, in the multivariable logistic regression analysis, only ITN ownership and the number of under-five children in the household were found to be significant predictors of malaria infection during high transmission season. Schoolchildren who did not have an ITN at home were 2.2 times more likely to be infected with malaria compared to those who had an ITN [AOR 2.2 (95% CI 1.2, 3.99)]. The odds of malaria infection were higher among schoolchildren living in households with one under-five child compared to those living in households without any under-five children [AOR 1.9 (95% CI 1.1, 3.38)] (Table 4).

**Table 4:** Factors associated with malaria infection among schoolchildren during high transmission season in Eastern Ethiopia

Variable	Positive	Negative	COR (95% CI)	p-value	AOR (95% CI)	P-value
School location						
Rural	9	303	1.0			
Peri-urban	16	306	1.6 (0.72, 3.58)	0.25		
Urban	23	291	2.4 (1.1, 5.13)	0.03		
Residence of the child						
Rural	10	308	1.0			
Peri-urban	16	308	1.6 (.72, 3.6)	0.25		
Urban	22	284	2.38 (1.1, 5.12)	0.03		
ITN ownership						
Yes	24	612	1.0	1.0		
No	24	288	2.13 (1.2, 3.8)	0.01	2.2 (1.2, 3.99)	0.01*
Slept under ITN last night						
Yes	18	402	0.74 (0.41, 1.4)	0.23		
No	30	498	1.0			
Number of under five children in the HH						
No child	17	388	1.0		1.0	
One child	23	300	1.75 (0.92, 3.3)	0.09	1.9 (1.1, 3.38)	0.035*
Two children	6	182	0.75 (0.3, 1.96)	0.56	0.83 (0.3, 2.2)	0.7
3 and above	2	30	1.52 (0.34, 6.9)	0.59	1.4 (0.31, 6.7)	0.65
Number of bed rooms						
One	11	282	1.0			
Two	27	373	1.86 (0.91, 3.8)	0.09		
Three	7	178	1.01 (0.38, 2.6)	0.98		
Four and above	3	67	1.15 (0.31, 4.3)	0.84		

In the low transmission season, school location, grade level, sex, family size, marital status of the household head, ITN ownership, and history of malaria infection were identified as candidate variables for the final model. However, only the marital status of the child’s household head was statistically significant. Children from households where the head was unmarried were 4.4 times more likely to be infected with malaria compared to those from households where the head was married (AOR 4.4; 95% CI 1.4–13.8) (Table 5).

**Table 5:** Factors associated with malaria infection among schoolchildren during low transmission season in Eastern Ethiopia

Variable and category	Positive	Negative	COR (95% CI)	p-value	AOR (95% CI)	P-value
School location						
Rural	11	336	1.0			
Peri-urban	4	318	0.6 (0.18, 2.1)	0.42		
Urban	7	307	1.7 (0.66, 4.5)	0.27		
Grade						
< = 3	10	598	1.0			
4–5	11	346	1.9 (0.8, 4.5)	0.15		
> = 6	1	17	3.5 (0.4, 29.1)	2.4		
Sex						
Male	8	550	1.0			
Female	14	411	2.3 (0.98, 5.6)	0.06		
Family size						
< 6	16	525	1.0			
6 and above	6	436	0.5 (0.17, 1.2)	0.1		
HH head marital status						
Unmarried	4	38	5.4 (1.74, 16.7)	0.003	4.4 (1.4, 13.8)	0.011
Married	18	923	1.0		1.0	
ITN ownership						
Yes	16	567	1.0			
No	6	394	0.5 (0.21, 1.4)	0.2		
Had history of malaria infection (in the last 6 months)						
Yes	4	348	0.4 (0.13, 1.2)	0.09		
No	18	613	1.0			

## Discussion

The present study investigated and compared the prevalence of malaria in urban, peri-urban and rural school children during high and low malaria transmission seasons in Eastern Ethiopia where urban malaria vector, *An. stephensi*, is widely spread. Accordingly, the prevalence of malaria among school children during high transmission season was 5.1% and the prevalence during low transmission seasons was 2.24%. The prevalence of malaria among schoolchildren during high transmission in urban, peri-urban and rural were 7.3%, 4.97%, 2.9% (P = 0.007), respectively. Similarly, the prevalence during low transmission season in urban, peri-urban and rural were 3.5%, 1.2%, and 2.04% (p = 0.3), respectively.

In this study the prevalence of malaria was high in urban area compared with peri-urban and rural areas. This is in line with the finding from North west Ethiopia [15] which found high malaria prevalence in urban setting. However, it is in contrast with the finding from Gabon [14] and Democratic Republic of Congo [17] which reported higher malaria prevalence in rural schoolchildren than urban. This might be due to the difference in ecological, climatic condition and the spread of the urban malaria vector *An. stephensi* in the Horn of Africa, including Ethiopia, which has increased the burden of urban malaria infections.

The prevalence during high transmission season is in line with the finding from Northwestern Ethiopia (6.8%) [15] and south west Ethiopia (5.03%), despite the use of qPCR for diagnosis [9]. Similarly, the prevalence during the low transmission season aligns with the microscopic finding from south-western Ethiopia (1.5%) [9]. However, those prevalences during both seasons are low compared to the prevalence of schoolchildren reported from Yemen (12.8%) [13], Democratic republic of Congo (33%) [17], and two studies from Tanzania (38.1%) [12] 26.4% [25]. The difference might be due to the difference in study setting, individual immunity, diagnostic method employed and seasonal variation.

Plasmodium falciparum both during high transmission (83.3%) and during low transmission season (59.1%) was the dominant species in this study which is similar with the finding from south western Ethiopia (90%) [9], Tanzania 38.1% [12], Yemen (96.7%) [13] and Iringa District of Tanzania (93.1%) [23]. The observed similarity may be attributed to shared ecological and climatic conditions that favor *P. falciparum* transmission, as well as comparable malaria control interventions and diagnostic practices across these regions. However, the finding from schoolchildren in Tanzania showed that *P. falciparum* occurred at a similar prevalence to other *plasmodium* species [30].

Moreover, this study identified multiple risk factors associated with malaria infection among schoolchildren, providing valuable insights for malaria control programmes to design targeted interventions. Accordingly, during low transmission season, marital status of the head of the household had statistically significant association with malaria infection among schoolchildren. Children whose parents were not in union were at risk of malaria infection. This is in line with the finding which analysed data from 11 sub-Saharan African countries that found children living with both parents had lower malaria prevalence compared to those living with others [31]. This suggests that household structure, which can be influenced by the marital status of the household head, may impact malaria risk among children. However, this finding contrasts with a study from Nigeria, which reported no significant difference in malaria infection rates between single and married individuals [35]. This discrepancy may be attributed to differences in study population and setting; while this study focused exclusively on school-aged children in a community-based context, the Nigerian study was conducted at a health facility level and included individuals from the general population.

On the other hand, schoolchildren without an insecticide-treated net (ITN) at home were more likely to be infected with malaria compared to those who had an ITN. This finding is in line with the finding from south western Ethiopia [9], Eastern Ethiopia [32] and Northwest Ethiopia [15]. This might be due to the fact that ITNs serve as a primary vector control intervention, reducing human-mosquito contact during peak biting hours and thereby lowering the risk of malaria transmission among children.

Schoolchildren from households with under-five children had higher odds of malaria infection compared to those from households without under-five children. Children under the age of five are particularly vulnerable to malaria due to their immature immune systems, which limit their ability to fight off infection. Their presence in the household may also increase malaria risk for older children, such as school-aged siblings, who often share sleeping spaces or stay nearby during nighttime care, increasing exposure to mosquito bites [3, 33–35].

There are several strengths of this study. It was conducted both during both high and low transmission seasons, allowing for a comprehensive understanding of malaria prevalence across different transmission periods. Additionally, the study examined the prevalence of malaria in urban, peri-urban, and rural schools, offering important insights into the disparities in risk and exposure in various contexts. However, the limitation of this study was self-reported data on ITN use may overestimate/underestimate actual usage, as children may not consistently follow recommended ITN practices, which could impact the reliability of these data.

## Conclusion

This study revealed that during high transmission season, malaria infection among schoolchildren residing in urban settings were high compared to peri-urban and rural areas. Similarly, during low transmission seasons, the prevalence was high in peri-urban followed by urban. The study also identified ITN ownership, marital status of the household head, and presence of children in the household as factors associated with malaria infection among schoolchildren. Hence, integrating schoolchildren, across both urban and rural schools, into malaria intervention strategies is crucial for strengthening control and elimination efforts. Furthermore, follow-up studies in the same schools and among the same children, along with the use of molecular diagnostic tools to detect low-density parasitaemias, are strongly recommended to enhance the effectiveness of malaria elimination programmes.

## Data Availability

The datasets used and/or analysed during the current study are available from the corresponding author on reasonable request.
